# YB-1 promotes microtubule assembly *in vitro* through interaction with tubulin and microtubules

**DOI:** 10.1186/1471-2091-9-23

**Published:** 2008-09-15

**Authors:** Konstantin G Chernov, Alain Mechulam, Nadezhda V Popova, David Pastre, Elena S Nadezhdina, Olga V Skabkina, Nina A Shanina, Victor D Vasiliev, Anne Tarrade, Judith Melki, Vandana Joshi, Sonia Baconnais, Flavio Toma, Lev P Ovchinnikov, Patrick A Curmi

**Affiliations:** 1 Institute of Protein Research, Russian Academy of Sciences, Pushchino, Moscow Region, 142290 Russia; 2 Laboratoire Structure-Activité des Biomolécules Normales et Pathologiques, INSERM/UEVE U829 Evry, 91025 France; 3 Department of Molecular Biology, Biological Faculty of Lomonosov Moscow State University, Moscow, 119991 Russia; 4 Dept. of Human Genetics, Hadassah University Hospital, Kiryat Hadassah, PO Box 12000, 91120 Jerusalem, Israel; 5 Laboratoire de Microscopie Moléculaire et Cellulaire, UMR 8126 CNRS-IGR-UPS, Institut Gustave-Roussy, 39 rue Camille Desmoulins, 94805 Villejuif, France

## Abstract

**Background:**

YB-1 is a major regulator of gene expression in eukaryotic cells. In addition to its role in transcription, YB-1 plays a key role in translation and stabilization of mRNAs.

**Results:**

We show here that YB-1 interacts with tubulin and microtubules and stimulates microtubule assembly *in vitro*. High resolution imaging via electron and atomic force microscopy revealed that microtubules assembled in the presence of YB-1 exhibited a normal single wall ultrastructure and indicated that YB-1 most probably coats the outer microtubule wall. Furthermore, we found that YB-1 also promotes the assembly of MAPs-tubulin and subtilisin-treated tubulin. Finally, we demonstrated that tubulin interferes with RNA:YB-1 complexes.

**Conclusion:**

These results suggest that YB-1 may regulate microtubule assembly *in vivo *and that its interaction with tubulin may contribute to the control of mRNA translation.

## Background

YB-1 is a multifunctional protein known to interact with nucleic acids, and as such, YB-1 is involved in a wide variety of cellular processes in eukaryotic cells (reviewed in [1]). In the cytoplasm, YB-1 participates in the formation of mRNPs and in the regulation of mRNA translation and degradation [2-6]. In the nucleus, YB-1 functions as a Y-box binding transcription factor, where it activates transcription of various cellular genes, including those implicated in cell growth, differentiation and apoptosis (reviewed in [7]). Translocation of YB-1 from cytoplasm to the cell nucleus can occur at certain steps of the cell cycle [8] and in response to stress-induced DNA damages [9,10]. The association of YB-1 with nucleic acids causes global changes in their structures by melting short or imperfect duplexes and acceleration of annealing and strand exchange reactions between complementary strands of RNA and DNA [11,12]. YB-1 may thus participate in DNA recombination and replication [13,14], and in the case of damaged DNA, YB-1 may assist in its reparation.

Molecular and structural investigations showed that YB-1 interacts with RNA and DNA through two non-homologous domains: the cold-shock domain (CSD), which consists of five anti-parallel beta-strands [15,16], and the C-terminal domain, which contains a series of alternating clusters of positively and negatively charged amino acid residues.

In addition to its interaction with nucleic acids, YB-1 interplays with different protein partners within the cell. It has been noticed that the interaction of YB-1 with p53 increases the affinity of p53 for DNA promoters that could stimulate transcription of p53-controled genes [17,18]. Similarly, YB-1 interacts with the T-antigen of polyomavirus JC and triggers transcription of viral genes [19]. YB-1 can also catalyze splicing of pre-mRNA via interaction with RNA polymerase II, EWS (Ewing's sarcoma protein) and TLS (translocation liposarcoma protein) proteins [20].

The objective of the present work was to identify new YB-1 protein partners to better understand the functions of this protein. We discovered, using a series of biochemical *in vitro *experiments, that YB-1 strongly interacts with tubulin, both soluble and polymerized into microtubules. We demonstrated that YB-1 stimulates microtubule assembly, and in addition, that tubulin competes with mRNA for interaction with YB-1. In light of these results, we propose that YB-1 may contribute to coordination of regulation of mRNA translation and dynamics of microtubule cytoskeleton. Tubulin, via its interaction with YB-1, may indirectly influence the translational regulation of mRNP complexes.

## Results

### Tubulin is a new YB-1 interacting protein

**Figure 1:**
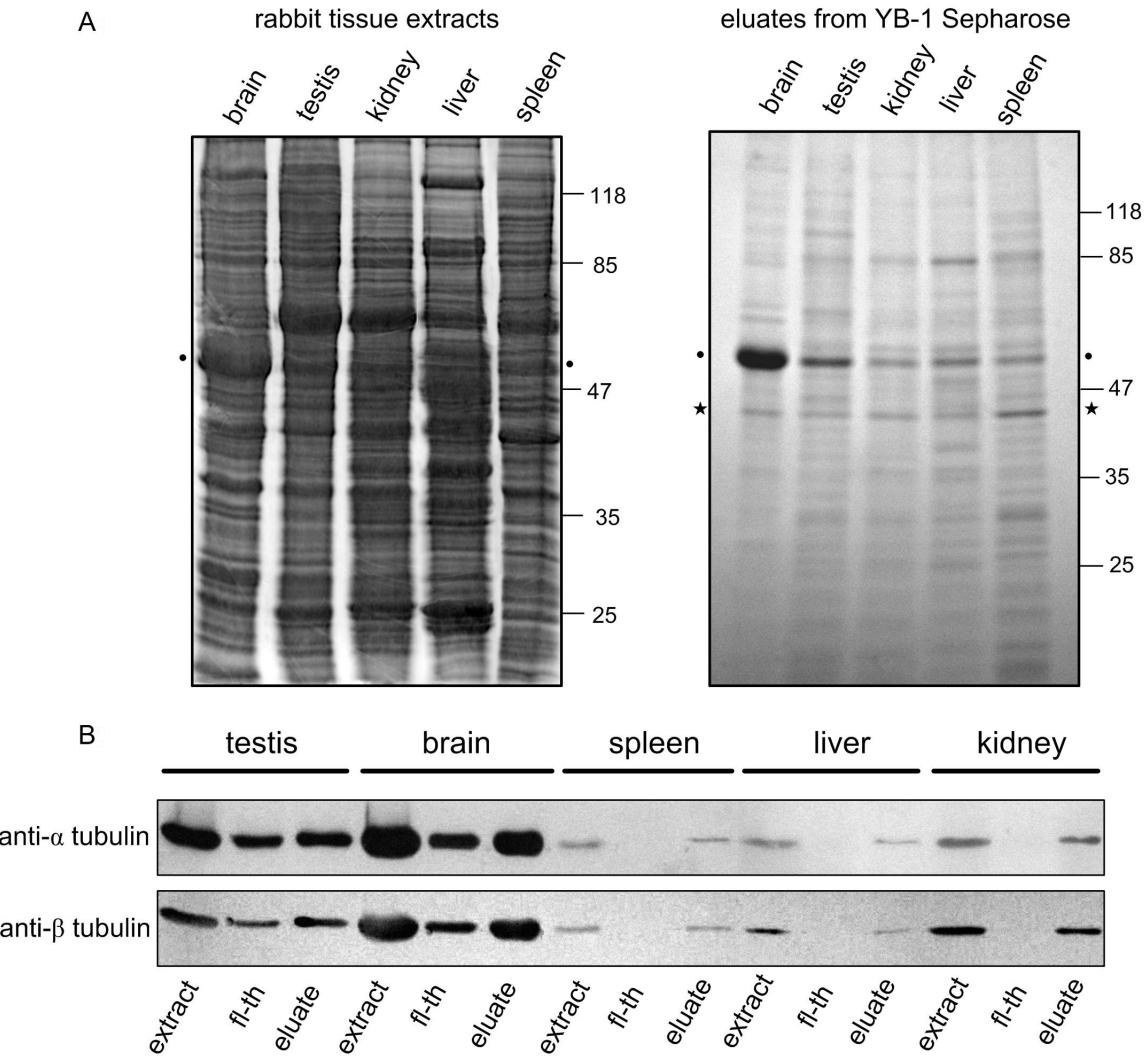
**YB-1 affinity chromatography of rabbit tissue extracts**. A, Rabbit tissue extracts (50 μg of total protein) were loaded onto YB-1-Sepharose. After washing with buffer containing 100 mM KCl, bound proteins were eluted with buffer containing 1 M KCl. Extracts and eluates were analyzed by 12% SDS-PAGE followed by Coomassie blue staining. Two prominent bands migrating as 45 kDa and 50 kDa were detected in the eluates of most of the tissues (marked with an asterisk and a dot) and were identified by mass spectrometry as actin and tubulin respectively. B, Western blot of rabbit tissue extracts and YB-1-Sepharose fractions. Fractions were collected as described in A and probed with anti-alpha and anti-beta tubulin antibodies (fl-th, flow through).

A search for new YB-1 partners from different rabbit tissue extracts was performed by affinity chromatography using YB-1-Sepharose as bait. Though eluates varied in protein composition, two prominent bands migrating as 45 kDa and 50 kDa were detected in the eluates of most of the tissues (Fig 1A, right panel, marked with an asterisk and a dot, respectively). The 45 kDa protein was identified by MALDI-TOF mass spectrometry as actin, a well-characterized YB-1 partner [21] and the 50 kDa band was identified by similar means as tubulin (see Additional file 1). The presence of tubulin in extract and eluate fractions was also confirmed by Western blotting using anti-alpha and anti-beta tubulin antibodies. As shown on Fig. 1B, α and β tubulin subunits were detected in all tissue extracts and high salt eluates. It is interesting to note that, in the case of brain and testis, some tubulin remained in the flow-through fraction. This could be either due to the saturation of the affinity column or linked to the sequestration of tubulin with partner proteins. In the control experiments performed with BSA-Sepharose, all tissue proteins were collected in the flow-through fractions and were undetectable in high salt eluates (data not shown). These results strongly suggested that tubulin binds specifically to YB-1.

**Figure 2:**
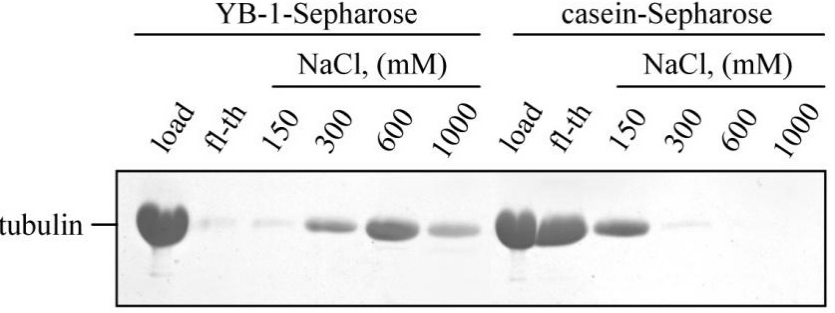
**YB-1 interacts directly with pure tubulin**. Affinity chromatography analysis of YB-1-tubulin interaction. Tubulin (20 μg) was applied onto 50 μl YB-1- or casein-Sepharose (used as a control) in buffer containing 150 mM NaCl at 4°C and incubated for 10 min. After washing, bound proteins were eluted stepwise using buffer containing 150 mM, 300 mM, 600 mM and 1 M NaCl as described under Materials and Methods. Fractions were analyzed by 15% SDS PAGE followed by Coomassie blue staining.

**Figure 3:**
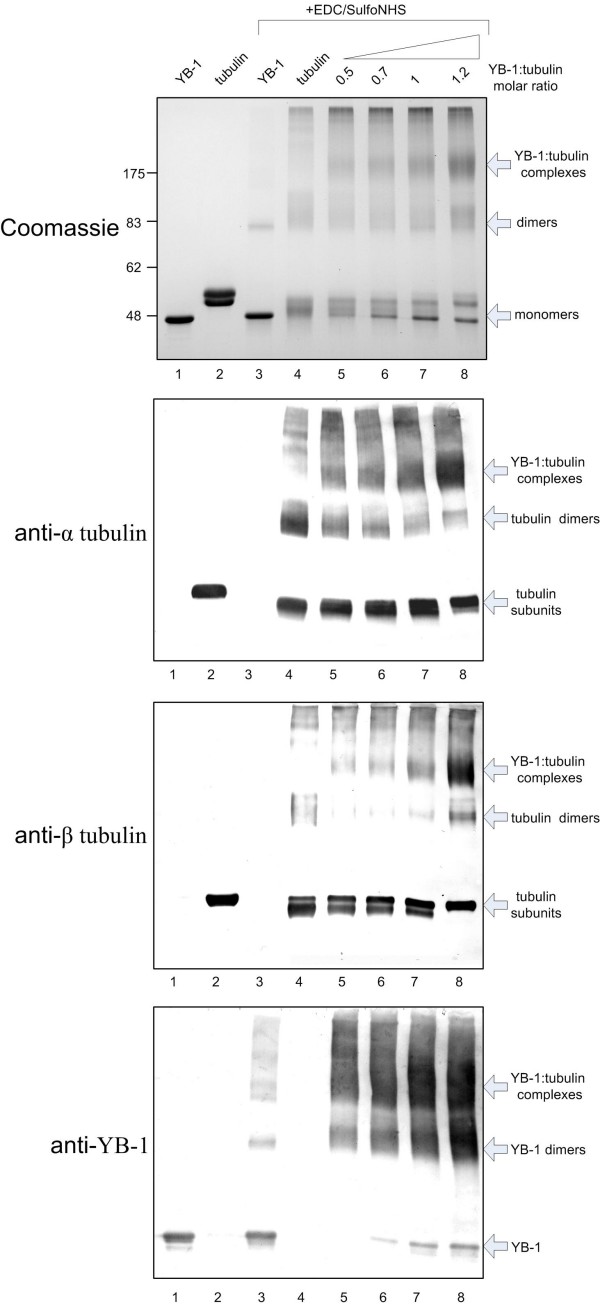
**YB-1:tubulin cross-linking**. YB-1 and tubulin were cross-linked with EDC and sulfo-NHS, resolved by 9% SDS-PAGE, blotted and probed with anti-alpha, anti-beta tubulin and anti-YB-1 antibodies as described under Materials and Methods. Gels were either stained with Coomassie blue (top) or processed for Western blotting with the indicated antibodies.

We further investigated YB-1:tubulin interaction and evaluated the stability of the YB-1-tubulin complex by YB-1 affinity chromatography using pure tubulin as a prey. Tubulin was totally adsorbed on the YB-1-Sepharose column in the presence of 150 mM NaCl, while poorly retained in similar conditions by a casein-Sepharose control column (Fig. 2). Tubulin started to elute from the YB-1 column at 300 mM NaCl forming a peak around 600 mM NaCl (Fig. 2). These results indicate that the YB-1:tubulin interaction is not inhibited at physiological and moderate ionic strength (up to 300 mM NaCl). We finally probed the presence of YB-1 and of α and β tubulin subunits in YB-1-tubulin complexes by cross-linking using the zero length cross-linker EDC/Sulfo NHS. As displayed on Fig. 3, cross-linker stabilized dimeric forms of YB-1 and α β-tubulin heterodimers, as well as higher molecular weight aggregates (compare lanes 3 and 4 with 1 and 2). It is worthy to note that under these conditions, both YB-1 and tubulin cross-linked samples still contained non-cross-linked products (lanes 3 and 4). When the cross-linking reaction was performed in the presence of both proteins at a YB-1-tubulin molar ratio of 0.5, most YB-1 was found in high-molecular weight complexes containing also both tubulin subunits (compare lanes 1 and 5). An increase of the YB-1:tubulin molar ratio over 0.5 increased the yield of these high molecular weight complexes (lanes 6 to 8) with a slight increase of the free YB-1 band. Our results demonstrate that YB-1 interacts directly with tubulin and suggest that YB-1 contacts the both tubulin subunits in solution.

**Figure 4:**
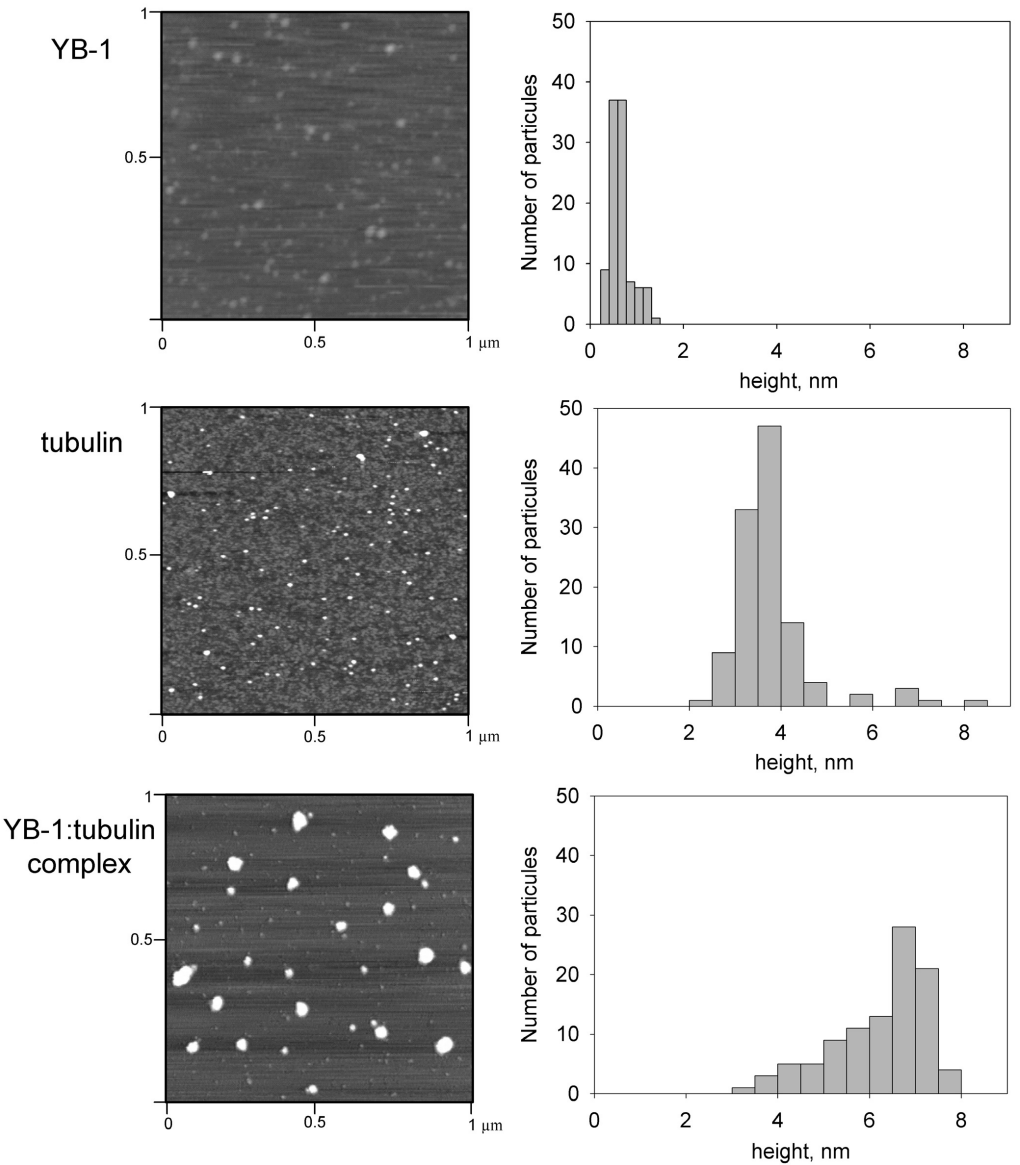
**AFM images of YB-1, tubulin and YB-1-tubulin complexes**. Isolated YB-1 and tubulin form discrete particles of 0.7 and 3.7 nm heights, respectively. YB-1-tubulin complex samples formed at a 1:1 molar ratio show the presence of an additional population of particles with a height of about 7.9 nm. Histograms illustrating the height distribution of particles are displayed on right panels.

The binding of YB-1 to tubulin was then investigated by AFM. YB-1 appeared as discrete particles on the mica surface with an average height of 0.7 nm (Fig. 4, upper panel). This value appeared lower than that reported by Skabkin et al [22] (about 4 nm for monomeric YB-1 in solution of high ionic strength) and probably resulted from YB-1 flattening on the mica surface. Tubulin appeared as particles with an average height of 3.7 nm (Fig. 4, middle panel) in agreement with previous reports [23]. In the YB-1-tubulin sample, in contrast to isolated proteins, particles were less homogenous with a size distribution ranging from about 3 to 8 nm (Fig. 4, bottom panel). A novel class of particles was clearly distinguishable, centered around 7 nm. It was attributed to the YB-1-tubulin complexes probably made of several molecules of both tubulin and YB-1.

### YB-1 strongly favors tubulin assembly *in vitro*

**Figure 5:**
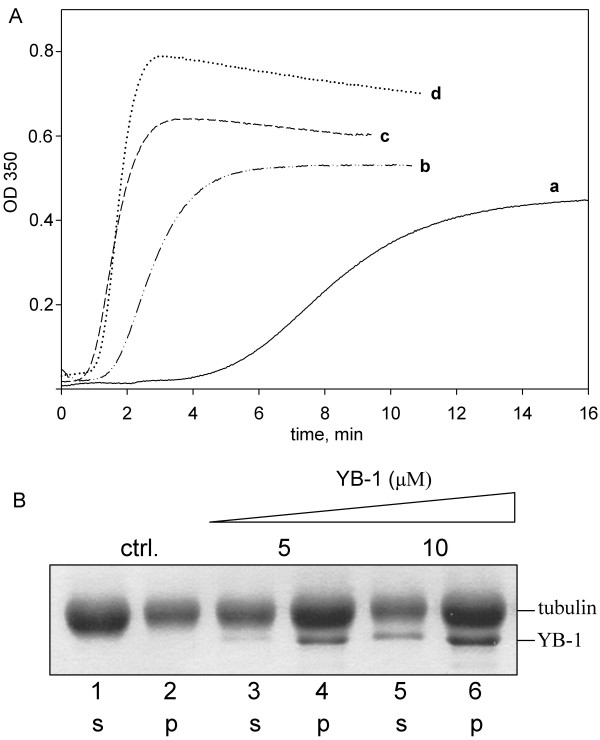
**YB-1 promotes microtubule assembly and co-sediments with microtubule pellets**. A, Turbidimetry plot of 20 μM tubulin assembly in the absence (a) or presence of increasing concentrations of YB-1 [2.5 μM (b), 5 μM (c) and 10 μM (d)] in buffer M with 10% glycerol. B, 20 μM tubulin was polymerized in buffer M with 10% glycerol in the absence (ctrl) or presence of 5 μM or 10 μM YB-1. After polymerization until steady-state, the samples were centrifuged, then supernatants and pellets were analyzed by SDS-PAGE. (S, supernatant; P, pellet). (Additional file 4).

The influence of YB-1 on *in vitro *microtubule assembly was first assessed by turbidimetry. Compared to tubulin control, YB-1 induced a dramatic shortening of the lag-time in a dose dependant manner. It also dose-dependently increased both the apparent rate of microtubule assembly and the steady state plateau value (Fig. 5A and Additional file 4). We next examined the distribution of YB-1 in the soluble tubulin or microtubule fractions in steady-state samples using microtubule sedimentation assay. In agreement with turbidimetry data, the presence of YB-1 strongly increased the total amount of tubulin in the pellet (Fig. 5B). In the presence of 5 μM YB-1 and 20 μM tubulin, most of YB-1 was associated with microtubules at steady state. In these conditions, a visual estimation on Coomassie stained gels of the YB-1:tubulin stoichiometry indicated a ratio of about 1 mole of YB-1 per 3 moles of tubulin heterodimer in the pellet. When 20 μM tubulin was assembled in the presence of 10 μM YB-1, we noted a further increase of the amount of tubulin in the pellet. YB-1 was again found mainly in the pellet with however a small amount remaining in the supernatant. In these conditions, the YB-1-tubulin stoichiometry in the pellet rose to about 1 mole of YB-1 per 2 moles of tubulin heterodimer. It is worthy to note that YB-1 alone, when centrifuged in the same conditions remained totally in the supernatant fraction (*data not shown*).

### YB-1 favors MAPs-tubulin assembly *in vitro*

**Figure 6:**
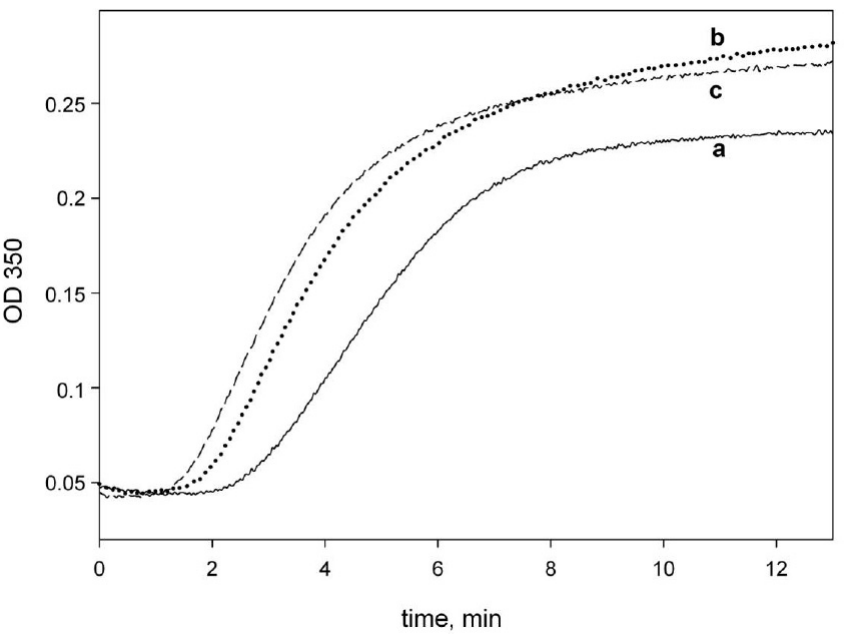
**YB-1 favors MAPs-tubulin assembly**. Turbidimetry plot of MAPs-tubulin (1 mg/ml, ~9 μM) assembly in buffer M with 20% glycerol in the absence (a) or presence of 2.5 μM (b), or 5 μM YB-1 (c).

It is well documented that the presence of MAPs strongly influences the kinetics of microtubule assembly. MAPs favor the nucleation of microtubules, increase the rate of assembly, extent of polymerization and stabilize microtubules against disassembly. To investigate whether YB-1 may also influence tubulin polymerization in conditions close to cellular, we performed a series of experiments with MAPs-tubulin. This preparation contained approximately 15% of MAPs (w/w) as estimated by Coomassie staining of proteins separated on SDS-PAGE. The addition of YB-1 to MAPs-tubulin at a total YB-1-tubulin ratio of 0.3 decreased the lag-time similarly to pure tubulin, increased the rate of polymerization and slightly increased the final microtubule mass as estimated from the steady state plateau value (Fig. 6). Higher amounts of YB-1 further reduced the lag-time of polymerization but did not change significantly the mass of polymerized tubulin. These data indicate that the presence of MAPs does not abrogate the positive effect of YB-1 on the overall kinetics of microtubule assembly.

### YB-1 accelerates tubulin S assembly *in vitro*

**Figure 7:**
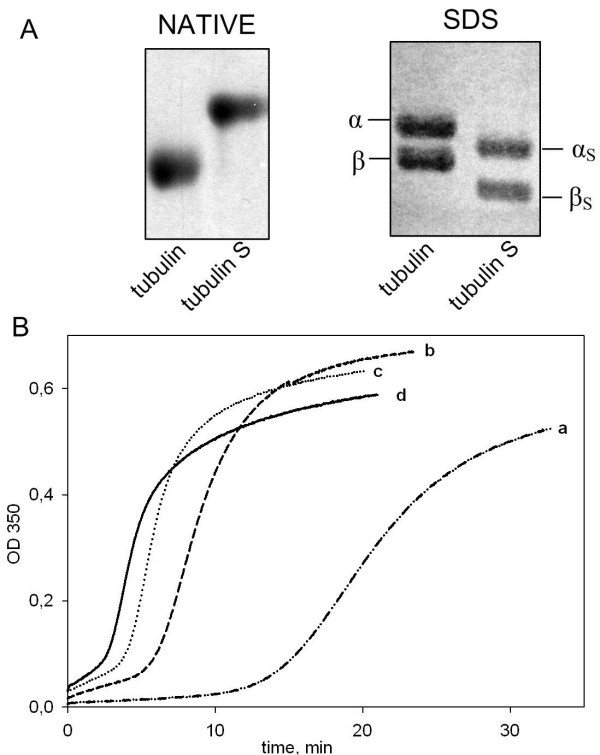
**YB-1 favors tubulin S assembly**. A, Tubulin and tubulin S (5 μM each) were analyzed by native agarose gel (left panel) and 8% SDS-PAGE (right panel). In our conditions, complete digestion of both α and β tubulin were total. B, Turbidimetry plot of tubulin S (5 μM) assembly in the absence (a) or presence of 0.625, 1.25, 2.5 μM YB-1 (b, c, and d, respectively) in buffer M with 20% glycerol.

The C-termini of alpha and beta tubulin subunits are highly negatively charged at physiological pH and involved in the regulation of tubulin function [24]. On the other hand, YB-1 is highly positively charged at neutral pH, so it was reasonable to think that YB-1 accelerates microtubule assembly via interaction with tubulin C-termini. It was therefore critical to investigate whether the effects of YB-1 on microtubule assembly could result only from a charge effect or could also be partly due to more specific molecular recognition mechanisms between YB-1 and tubulin. For this purpose, we treated tubulin with subtilisin in such conditions as to cleave the charged C-termini of the both tubulin subunits and investigated the effect of YB-1 on the assembly of the cleaved tubulin product (tubulin S, Fig. 7A). We observed that YB-1 was still able to promote the assembly of tubulin S (Fig. 7B). A strong stimulation of assembly was observed when YB-1 was added at a total YB-1-tubulin S molar ratio as low as 0.13. At this ratio, YB-1 significantly decreased the lag-time and increased the velocity of the polymerization (Fig. 7B, curve b). Higher concentrations of YB-1 further reduced the lag-time and increased the rate of tubulin S assembly but did not produce any significant additional effect on microtubule mass at steady-state (Fig. 7B, curves c and d). Together, these data suggest that the promotion of microtubule assembly by YB-1 involves interaction between specific sites of the partners, although non-specific electrostatic interactions may also play an important role.

### YB-1 promotes the formation of normal microtubules and probably coats the microtubule wall

**Figure 8:**
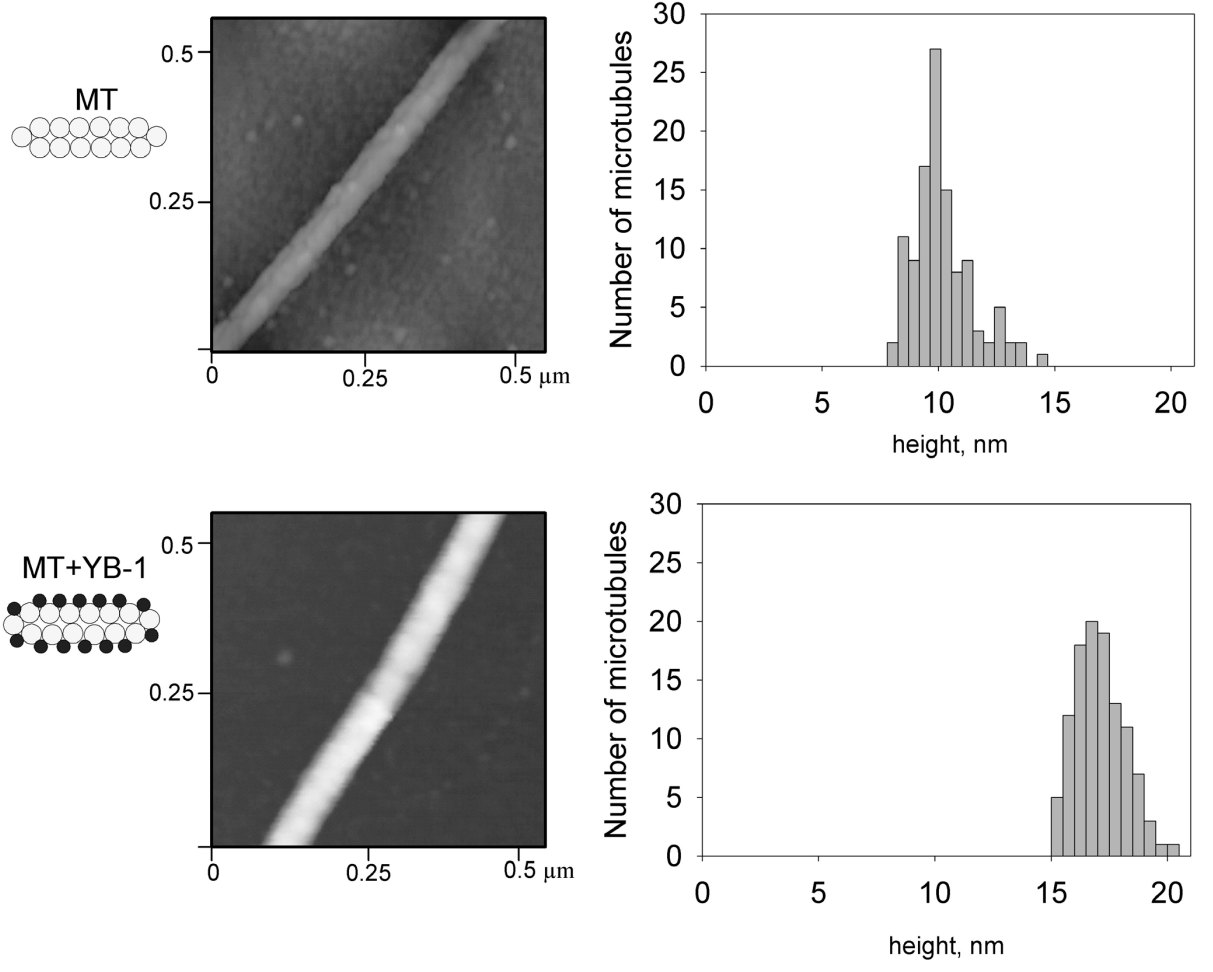
**AFM images of microtubules assembled with or without YB-1**. Microtubules were assembled with (bottom panel) or without YB-1 (top panel), fixed and analyzed by AFM as described under Materials and Methods. Histograms of the microtubule height distribution are shown on the right. Schemas on the left illustrate the proposed structure of microtubules.

High resolution microscopies like AFM or TEM could provide interesting information about microtubule morphology in the presence of YB-1 and about the localization of YB-1 on/in microtubules. Under control conditions, i.e., without YB-1, in AFM images microtubules appeared as straight rods with an apparent height of 10 nm. This indicates that they were flattened on the surface due to the drying procedure (Fig. 8, upper panel) and the height measurement thus corresponded to two tubulin layers in close contact (see [23] and Fig. 8, upper schema). Microtubules formed in the presence of YB-1 were significantly higher than the control ones with an average height of about 17 nm. The increase in height could correspond to YB-1 coating of the microtubule outer wall (Fig. 8, bottom panel and schema). However, such morphology can also result from microtubules with double walls or with a higher number of protofilaments.

**Figure 9:**
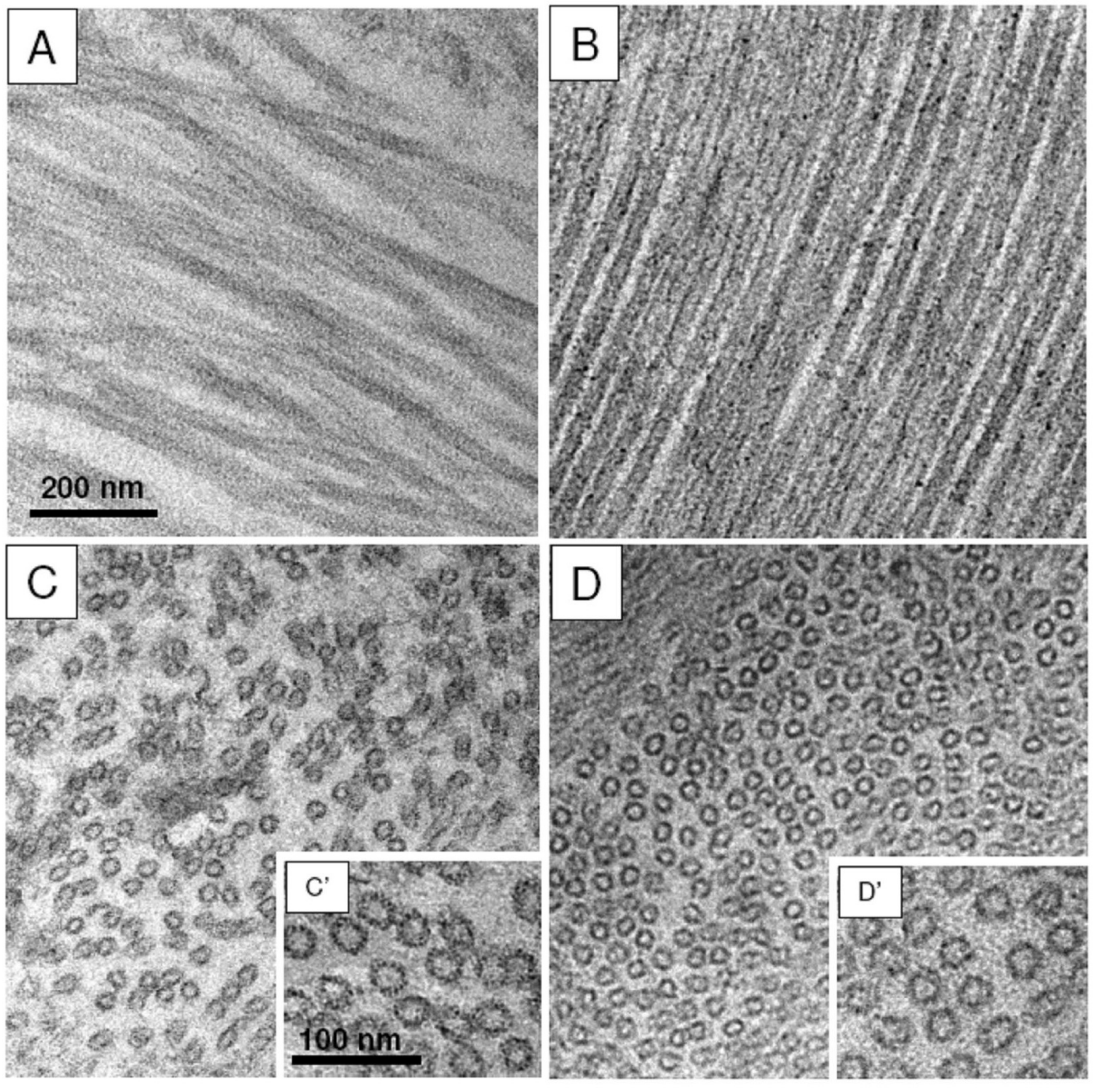
**YB-1 induces the formation of single wall microtubules**. Tubulin (30 μM) was polymerized without (A, C, C') or with 10 μM YB-1 (B, D, D') at 37°C. Transmission electron microscopy of longitudinal ultrathin sections revealed that YB-1 contributes to the formation of normal microtubules and that, in addition, the microtubules remain regularly spaced from one another (compare B with the control A). Transversal sections of microtubules (C, D) confirmed this observation. B, C, D are at the same magnification as A. D' is at the same magnification as C'.

To distinguish between these scenarios, we investigated the effect of YB-1 on the ultrastructure of microtubules by TEM with thin sections of EPON-embedded microtubules. In control conditions, TEM analyses showed regular microtubules with a diameter of about 25 nm. With YB-1, the ultrastructure of microtubules remained normal with a single layer of tubulin forming their wall and an outer diameter comparable to control (Fig. 9, compare D' with C'). In addition to this, the number of protofilaments was not significantly different from that of control (see Additional file 2). This supported further the suggestion that YB-1 coats the outer surface of the microtubule. It is also worth noting that, under control conditions, microtubules could often be found in close contact with each other (Fig. 9A, C and 9C'), whereas in the presence of YB-1, microtubules appeared regularly distributed and spaced from each other (Fig. 9B, D and 9D'). Due to its coating the microtubule surface, YB-1 could induce a change in rigidity of microtubule or steric hindrance on its outer surface, which may lead to a larger inter-microtubule spacing.

### Tubulin interferes with mRNP formation

**Figure 10:**
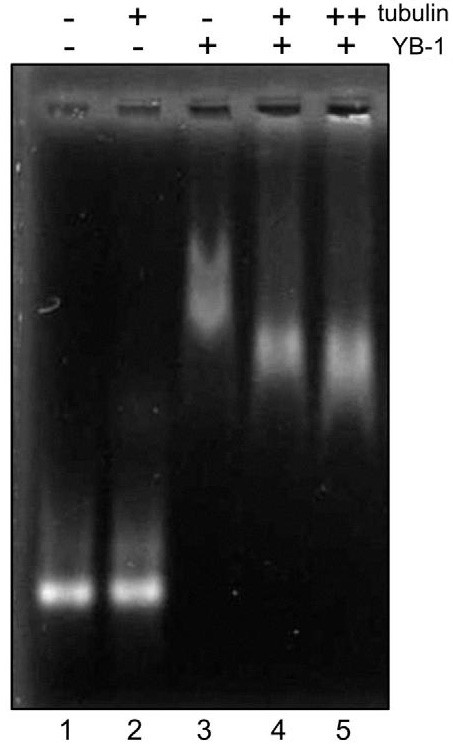
**Tubulin modifies the structure of YB-1-RNA complexes**. 30 nM Luciferase RNA was incubated in buffer M with 5% glycerol alone (lanes 1 and 2) or in the presence of 2.1 μM YB-1 (lanes 3, 4 and 5) for 10 min at 25°C. After incubation, 2.1 μM tubulin (lane 4) or 4.2 μM tubulin (lanes 2 and 5) were added and mixtures were analyzed by native agarose gel electrophoresis followed by staining with ethidium bromide.

YB-1 is a major mRNA binding protein that forms complexes with mRNA and regulates its translational activity. In this context, it is necessary to explore whether tubulin can interfere with formation of RNP and induce some modifications of RNP structure. Since RNP differs from naked RNA in net charge and molecular weight, we decided to investigate the effect of tubulin on YB-1-RNA complexes by electrophoretic mobility gel-shift assay. It is known that RNP saturated with YB-1 contains about one molecule of YB-1 per 25 RNA bases [25]. Compared to RNA alone, these saturated YB-1:RNA complexes demonstrate a significant reduction of mobility in native agarose gel due to either partial RNA discharging or the increased mass of the formed YB-1:RNA complex, or both (Fig. 10, compare lane 1 and 3). The presence of tubulin did not change mobility of RNA that excluded the possibility of direct interaction between these two molecules (compare lanes 1 and 2). The addition of tubulin to YB-1:RNA complexes clearly increased the mobility of RNP (compare lane 3 with 4 and 5). The presence of tubulin may thus induce structural changes of RNP complexes and/or a change in the RNP charge via YB-1 withdrawal mediated by tubulin. Further investigations are required for a better description of the interaction between mRNA:YB-1 complexes and tubulin, which may play an important role in transition of mRNA from silenced to translationally active state.

## Discussion

YB-1 is a member of the Y-box protein family, a family highly conserved among prokaryotes and eukaryotes. In eukaryotes, Y-box proteins are regulators of transcription and translation and play important roles during the development and in cell proliferation and transformation. Here, we report that YB-1 interacts with tubulin and microtubules *in vitro *and demonstrate that this protein promotes the assembly of microtubules. This new functional link of cellular proteins seems to be important, since, on the one hand, the regulation of many vital processes, such as cell division, motility and intracellular traffic depends on the fine tuning of the intrinsic dynamics of microtubules by protein partners. The inventory of tubulin partners is not complete and the comprehension of their mechanisms of regulation of microtubule dynamics is still under investigation. On the other hand, with YB-1 being an RNA-binding protein, the study of YB-1:tubulin interaction is highly relevant to further address the competition between tubulin and mRNA for YB-1 binding. The conformational changes of RNP containing YB-1 induced by tubulin may be of particular interest to investigate RNP accessibility for translation and degradation.

### YB-1 strongly interacts with tubulin

The strong binding between YB-1 and tubulin was supported by a series of *in vitro *biochemical data which also demonstrated that the YB-1:tubulin interaction was independent of the presence of other tissue extract components. Since YB-1 is positively charged at neutral pH (pI ~ 9.5) [2] while tubulin is negatively charged (pI ~ 5.6) [26], we assayed if YB-1 interacts with tubulin in a manner similar to that of many other positively charged nucleic acid binding proteins. As tubulin was still retained on a YB-1 column at physiological ionic strength, we concluded that the attraction between tubulin and YB-1 was the result of a strong, short-ranged, electrostatic interactions and (or) other more specific mechanisms like hydrophobic interactions. Additionally, we found that YB-1 did not provoke the formation of huge aggregates containing tubulin, in contrast to other highly cationic proteins like histones [27]. The YB-1-tubulin oligomeric complexes formed in "non-assembly conditions" had their heights about 7 nm (Fig. 4), which indicated the involvement of a few YB-1 and tubulin molecules in formation of these complexes.

Microtubule polymerization is a two-step process in which tubulin first forms nucleation templates and then adds to and elongates them [28,29]. The templates were modelled either in equilibrium with tubulin dimers [30-32] or as persistent structures [33]. MAPs facilitate the formation of templates and are thought to clamp them. The YB-1-tubulin complexes we observed here were functionally reminiscent of MAPs-tubulin complexes [29]. The YB-1-tubulin complexes could represent nucleation templates, and thus, YB-1 could act as a nucleation factor for microtubule assembly *in vitro *in a manner similar to that of classical MAPs [34].

### YB-1 favours microtubule assembly and coats the microtubule wall

YB-1 strongly favoured polymerization of pure tubulin into microtubules. YB-1 was found to accelerate the apparent rate of microtubule assembly, to increase the microtubule mass at steady state and to be associated with microtubules.

The ability of MAPs or other non-specific basic molecules to promote microtubule assembly depends largely upon the presence of the negative charges of tubulin C-termini [35,36]. In contrast to these positively charged molecules, we found that YB-1, which is also a basic protein, still stimulated polymerization of tubulin cleaved by subtilisin. This observation suggests that the promotion of microtubule assembly by YB-1 results not only from purely electrostatic interactions but also from a more specific molecular recognition mechanism. This suggestion is supported by the fact that most positively charged molecules which interact in a non-specific manner with tubulin promote the assembly of tubulin into aberrant structures instead of microtubules. For example, polycations induce the formation of double-walled microtubules [37,38], and aminoacyl-tRNA synthetases promote microtubule bundling [39]. In the present work, electron microscopy analysis showed that microtubules formed in the presence of YB-1 possess a normal morphology with a single wall of circa 25 nm diameter and parallel AFM investigations showed that YB-1 can coat the outer surface of the microtubule wall.

In agreement with these microscopic data, YB-1 was found to co-sediment with microtubules (Fig. 5). The stoichiometry corresponds well to some other microtubule-binding proteins with single tubulin dimer-binding site [40,41]. Many canonic MAPs were reported to bind microtubules with a stoichiometry ranging from 1:15 to 1:8 (MAPs:tubulin ratio in the microtubule wall) probably because of multiple tubulin-binding sites and high molecular mass of these proteins [42].

### YB-1 promotes polymerization of MAPs-tubulin

In the presence of MAPs-tubulin, YB-1 also accelerated microtubule assembly, although it didn't promote an increase of the total mass of polymerized tubulin. This was most probably due to the fact that our MAPs-tubulin preparation contained around 15% of MAPs (w/w), which makes microtubules nearly saturated with MAPs. Interestingly, some high molecular weight MAPs were partially displaced by YB-1 from MAPs-microtubules stabilized with taxol when the total YB-1-tubulin molar ratio was above 0.5 (see Additional file 3). As mentioned above, classical MAPs, such as tau and MAP2, interact mostly with the negatively charged C-termini of tubulin. In addition, tau binds to the amino terminal region of alpha tubulin subunit [43]. Since YB-1 strongly promoted assembly of tubulin lacking C-termini, we predict that YB-1 interacts with microtubules not only via non-specific binding to the negatively charged C-terminal tail. Finally, in this context, it is interesting to note that YB-1 doesn't display any homology with classical MAPs and may represent the generic element of a novel class of microtubule interacting proteins.

### YB-1 may shuttle between mRNA and the microtubule cytoskeleton *in vivo*

Microtubules play a critical role in mRNA translation and localization *in vivo *(reviewed in [44]). Numerous plant proteins known to regulate translation were recently identified by affinity chromatography as tubulin-binding proteins [45]. For example, the plant initiation factor eIF-(iso)4F was found associated with the microtubule cytoskeleton in the cell and is able to induces microtubule bundling *in vitro *[46].

Since YB-1 is a major component of cellular RNPs, interaction of tubulin with YB-1 could potentially regulate the translation state of mRNAs. Indeed, YB-1 protects mRNAs from degradation and packs them in non-translatable RNPs. We have shown here that tubulin changes the electrophoretic mobility of YB-1-RNA complexes. This change most probably occurs due to the partial dissociation of YB-1 from RNA, which could be a signal to trigger mRNA translation and/or degradation. Interestingly, it has recently been shown that the phosphorylation of YB-1 by Akt kinase may regulate its binding to mRNA [47]. It is thus expected that the phosphorylated form of YB-1, which possesses a lower affinity for the cap-structure of mRNA, could be more easily released from RNA than its non-phosphorylated form. This release could occur due to interaction of mRNA with other RNA-binding proteins or through the interaction of YB-1 with protein partners, such as tubulin. Our results thus provide new prospects on the role of protein-protein interaction in the regulation of mRNA translation. Furthermore, these results could also help to unravel situations where tubulin is involved in the overall processes of mRNA translation and degradation.

## Conclusion

Thus, we have first demonstrated that YB-1 stimulates microtubule assembly *in vitro*. By all *in vitro *criteria, YB-1 represents a novel microtubule-interacting protein related to the function of MAPs but clearly with different properties. The YB-1 properties described here may contribute to the understanding of its role in the cell division and embryogenesis and shed light on its oncogenic and anti-oncogenic activities [48-51]. Finally, these results could provide a framework to bridge different aspects of regulation of mRNA translation and the function of the microtubule cytoskeleton.

## Methods

Unless stated otherwise, chemicals were purchased from Sigma-Aldrich (Milwaukee, WI, USA).

### YB-1 purification

YB-1 was expressed in the *Escherichia coli *BL21(DE3) strain transformed with the pET 3-1-YB-1 construct [12]. Bacteria were cultured at 37°C to mid-log exponential phase, then protein synthesis was induced by 0.5 mM IPTG. After 3 h induction, bacteria were pelleted by centrifugation (4000 × g, 10 min) and the pellet was resuspended in 10 volumes of 40 mM Tris-HCl, pH 7.6, 2 M NaCl, 1 mM PMSF and disrupted by ultrasonication. Cell debris was removed by centrifugation at 140 000 × g for 2 h. Supernatant was diluted with four volumes of 10 mM Tris-HCl, pH 7.6, and loaded onto a heparin-Sepharose column (GE Healthcare, UK) equilibrated with 20 mM Tris-HCl, pH 7.6, 500 mM NaCl. The column was washed with 5 volumes of 20 mM Tris-HCl, pH 7.6, 500 mM NaCl, after that bound YB-1 was eluted with 20 mM Tris-HCl, pH 7.6, 2 M NaCl. Eluted protein was concentrated by centrifugation using a Centriprep 10 concentrator (Amicon Corporation, Danvers, MA) and purified by size exclusion chromatography on a Sephacryl S-200 column (GE Healthcare) pre-equilibrated with 20 mM Tris-HCl, pH 7.6, 2 M NaCl. Fractions containing YB-1 were pooled, dialyzed against 20 mM Tris-HCl, pH 7.6, 250 mM NaCl and concentrated again using the same procedure. Protein concentration was determined by comparison with a standard BSA curve using the Bio-Rad protein assay kit (Bio-Rad Laboratories, Richmond, CA). Anti-YB-1 antibodies were produced in rabbit as described by Davydova et al. [52].

### Tubulin and microtubule proteins preparation

Tubulin was purified from sheep brain using the method of Castoldi and Popov [53] and stored at -80°C in 50 mM MES-KOH, pH 6.8, 0.5 mM dithiothreitol, 0.5 mM EGTA, 0.25 mM MgCl_2_, 3.4 M glycerol, 0.1 mM GTP. Before use, tubulin stock was thawed and an additional cycle of polymerization was performed. Microtubule proteins (tubulin + microtubule-associated proteins, MAPs, herein referred to as MAPs-tubulin) were purified from sheep brain through two cycles of assembly/disassembly as described by Mitchison and Kirschner [54] aliquoted and stored at -80°C. Before use, MAPs-tubulin preparation was rapidly thawed and centrifuged at 25 000 × g 10 min to remove aggregated material.

### Tubulin S preparation

Tubulin S was prepared as described by Knipling et al. [55]. Briefly, 250 μM tubulin in the stock buffer was diluted five times with 1 mM GTP in water. Subtilisin was then added to reach a subtilisin/tubulin ratio of 1/200 (w/w). The mixture was incubated for 40 min at 25°C, and PMSF was added to 0.5 mM to stop cleavage. We then added MES-KOH, pH 6.8, MgCl_2 _and EGTA to reach a final concentration of 50 mM, 1 mM and 1 mM of these compounds, respectively. The mixture was incubated on ice for 30 min and centrifuged at 100 000 × g for 10 min. Supernatant was collected and used immediately for polymerization assays.

### Rabbit tissue extracts preparation

Tissue extracts from adult rabbit were prepared as described by Miwa et al [56]. Briefly, tissues were homogenized in 3 volumes of 50 mM Tris-HCl, pH 7.6, 50 mM KCl, 5 mM MgCl_2_, 0.25 M sucrose, 1 mM DTT, 1 mM PMSF in motor-driven homogenizer, and homogenates were centrifuged at 10 000 × g for 20 min to remove cell debris. Supernatants were collected and protein concentration was determined as described above.

### Affinity chromatography

YB-1, BSA and casein were coupled to Sepharose 4B using 5 mg of protein per 1 ml of CNBr-activated Sepharose 4B (GE Healthcare) according to the manufacturer's instructions.

Rabbit tissue extracts (50 μg of total protein) were incubated with 10 μl of YB-1- or BSA-coupled Sepharose in 50 μl of low salt buffer (20 mM Tris-HCl, pH 7.6, 100 mM KCl, 1 mM MgCl_2_, 1 mM DTT, 1 mM PMSF) for 10 min at room temperature. Reaction mixtures were centrifuged at 1 500 × g for 1 min, supernatants were discarded, and the resin pellets were resuspended in 250 μl of incubation buffer and pelleted again. This washing step was repeated twice. Bound proteins were eluted with 250 μl of high-salt buffer (20 mM Tris-HCl, pH 7.6, 1 M KCl, pH 7.6, 1 mM MgCl_2_, 1 mM DTT, 1 mM PMSF) and precipitated by addition of 75% acetone (v/v). Dried pellets were dissolved in SDS sample buffer and analyzed by electrophoresis on 12% SDS-PAGE. For the identification of tubulin, proteins were separated similarly by SDS-PAGE and then blotted on nitrocellulose as described [57]. Nitrocellulose membranes were blocked with 1% nonfat milk in TBST buffer (10 mM Tris-HCl, pH 7.6, 140 mM NaCl, 0.1% Triton X-100) and probed for tubulin with mouse anti-alpha (clone B-5-1-2) and anti-beta tubulin antibodies (clone Tub 2.1) diluted at 1/5000 in blocking solution. Primary antibodies were detected using goat anti-mouse horseradish peroxidase conjugated secondary antibodies followed by development using 3,3',5,5'-tetramethylbenzidine (TMB).

### Analysis of YB-1:tubulin complex stability

Tubulin (20 μg) was loaded onto 50 μl of YB-1- or casein-coupled Sepharose 4B columns equilibrated with 10 mM Tris-HCl, pH 7.6, 150 mM NaCl and incubated for 10 min at 4°C. Flow-through was collected, and the resins were washed with five column volumes of equilibrium buffer. Bound tubulin was eluted stepwise using five column volumes of 10 mM Tris-HCl, pH 7.6, containing either 300 mM, 600 mM or 1 M NaCl. Proteins from all fractions were precipitated by addition of trichloroacetic acid to 10% and analyzed by SDS-PAGE.

### *In vitro* tubulin polymerization assays

Tubulin or MAPs-tubulin assembly was followed turbidimetrically at 340 nm (1 cm light path) in an Ultrospec 3000 spectrophotometer (GE Healthcare) equipped with a temperature controller. Experiments were carried out in buffer M (50 mM MES-KOH pH 6.8, 1 mM EGTA, 5 mM MgCl_2 _and 1 mM GTP) with either 10 or 20% glycerol (v/v).

### Microtubule sedimentation assays

50 μl of 25 μM tubulin in buffer M containing 10% glycerol were assembled at 37°C with increasing concentrations of YB-1 for 30 min to reach the steady-state. Microtubules were pelleted at 300 000 × g for 5 min at 37°C and resuspended in 50 μl of SDS sample buffer. To determine the amounts of tubulin and YB-1 in the microtubule or supernatant fractions, 3 μl of supernatants and resuspended pellets were analyzed by SDS-PAGE.

### Cross-linking of YB-1:tubulin complexes

Cross-linking reactions were performed at tubulin concentration far below the critical concentration. To prepare YB-1:tubulin complexes, 1 μM tubulin and 0.3, 0.5, 0.8 or 1.2 μM YB-1 were incubated in 50 mM MES-KOH, pH 6.8, 1 mM MgCl_2_, 1 mM EGTA, 0,5 mM GTP, 5 mM EDC, 12 mM sulfo-NHS for 1 h at 30°C. Cross-linking reactions were quenched by the addition of 50 mM glycin. Proteins were precipitated with 75% acetone, dissolved in SDS sample buffer and resolved on 9% SDS-PAGE. Protein detection was performed using rabbit anti-YB-1 antibodies [52], anti-alpha tubulin antibodies and anti-beta tubulin antibodies as described above.

### Atomic Force Microscopy (AFM)

To study YB-1:tubulin complexes, we prepared reaction mixtures with either both or separately taken tubulin and YB-1 (1 μM each) in 50 mM MES-KOH, pH 6.8, 1 mM MgCl_2_, 1 mM EGTA, 0.5 mM GTP. These mixtures were incubated for 10 min at 37°C, then fixed with 0.2% glutaraldehyde.

Microtubules were prepared for AFM imaging as follows: 5 μM tubulin was assembled with or without (control) 5 μM YB-1 in 50 mM MES-KOH, pH 6.8, 1 mM EGTA, 5 mM MgCl_2_, 1 mM GTP, and 20 μM taxol for 15 min at 37°C, pelleted as described above, gently resuspended in a starting volume of polymerization buffer with 0.2% glutaraldehyde and fixed for 15 min at 37°C.

All AFM samples were deposited on Ni^2+ ^pretreated freshly cleaved mica as described by Pastre et al. [58]. AFM imaging was performed in tapping mode with a multimode AFM instrument (Digital Instruments, Veeco, Santa Barbara, CA) operating with a Nanoscope IIIa controller. We used AC160TS silicon cantilevers (Olympus, Hamburg, Germany) with a resonance frequency of 300 kHz. Images were collected at a scan frequency of 1.5 Hz and a resolution of 512 × 512 pixels.

### Transmission Electron Microscopy (TEM)

For ultrathin sectioning, microtubules were prepared with 30 μM tubulin with or without 10 μM YB-1 in 50 mM MES-KOH, pH 6.8, 1 mM EGTA, 5 mM MgCl_2 _and 1 mM GTP, 10% glycerol. Microtubules were pelleted at 40 000 × g for 30 min at 37°C. The pellets were gently resuspended in 50 mM MES-KOH, pH 6.8, 1 mM EGTA, 5 mM MgCl_2_, 1 mM GTP, 1% glutaraldehyde and incubated for fixation for 1 hour at room temperature. Samples were then post-fixed with 1% osmium tetraoxide (OsO_4_) for 1 h. After gradual dehydratation in ethanol series, the pellets were embedded in EPON mixture. Ultrathin sections were stained with 2% uranyl acetate and examined with a Tecnai F20 S-Twin transmission electron microscope (FEI company, Hillsboro, OR, USA) operating at 200 kV.

### Electrophoretic mobility shift assay

Luciferase RNA (1500 base length) was synthesized *in vitro *as described by Svetlov et al [59]. RNA (0.6 pmoles) was incubated in 20 μl of buffer M containing 5% glycerol alone or in the presence of 42 pmoles YB-1 for 10 min at room temperature. After incubation, either 42 pmoles or 84 pmoles tubulin were added to preformed RNP. The reaction products were separated at 80V for 2 hours on 0.6% agarose gel prepared in buffer M. After migration, the gel was stained with ethidium bromide.

## Abbreviations

MT: microtubules; MAPs: microtubule associated proteins; RNP: ribonucleoprotein; Tubulin S: subtilisin-treated tubulin; TEML: transmission electron microscopy; EDC: 1-ethyl-3-(3-dimethylaminopropyl) carbodiimide; Sulfo-NHS: N-hydroxysulfosuccinimide.

## Authors' contributions

KGC: Designed and performed experiments. Participated to the analysis of data, to the writing of the manuscript and revision. AM: Performed experiments, participated to the analysis of data and critical revision. NVP: carried out initial experiments on affinity chromatography of cell extracts and tubulin on YB-1-Sepharose, participated in experiments on YB-1 co-sedimentation with microtubules and in manuscript drafting. DP: Performed AFM investigation, participated to the revision of the manuscript. ESN: was involved in the study design and drafting the manuscript and in revising it critically for important intellectual content. OV: carried out the molecular genetic studies and YB-1 purification and affinity chromatography, coordinated studies on YB-1-tubulin interaction and on analysis of stability of this complex. NAS: isolated tubulin and obtained microtubules, participated in experiments on YB-1-microtubule interaction. VDV: participated in analysis and interpretation of AFM and TEM data and in revising the manuscript. AT: performed TEM. JM: participated to TEM. VJ: production and purification of recombinant protein. SB: participated to TEM. FT: participated to critical review of manuscript. LPO: Conceived of study, participated in design and coordination. PAC: Conceived of study, participated in design and coordination, participate to the writing and revision of the manuscript. All authors read and approved the final manuscript.

## Supplementary Material

Additional file 1 MALDI-TOF peptide scores for proteins found in eluates after chromatography of rabbit tissue extracts on YB-1-Sepharose. Click here for file

Additional file 4 Plot of the tangent at the microtubule assembly slope versus YB-1 concentration observed on figure 5. We can notice that rate of microtubule assembly reaches a maximum plateau value from about 8 μM YB-1. Click here for file

Additional file 2 Histogram of protofilament number observed in control microtubules and in the presence of YB-1. Click here for file

Additional file 3 YB-1 partially displaces MAPs from taxol-stabilized microtubules. MAPs-tubulin (0.5 mg/ml, ~4μM) was polymerized in the absence (control) or presence of increasing concentrations of YB-1 (from 1.25 μM to 10 μM, as indicated) in buffer M with 10% glycerol and 20 μM taxol. After polymerization, the samples were pelleted, and equal volumes of supernatants and resuspended pellets were analyzed by SDS-PAGE. (S, supernatant; P, pellet). Click here for file
