# Supplementary material for: YB-1 promotes microtubule assembly in vitro through interaction with tubulin and microtubules
Source: BMC Biochem. 2008 Sep 15;9:23. doi: 10.1186/1471-2091-9-23 (PMC2557009; doi:10.1186/1471-2091-9-23)
Supplement: Additional file 1 — MALDI-TOF peptide scores for proteins found in eluates after chromatography of rabbit tissue extracts on YB-1-Sepharose. [file 1471-2091-9-23-S1.doc]

| Eluate | *M*_r_, kDa | Accession no. | Score | Name of protein |
| --- | --- | --- | --- | --- |
| brain | 50 | AAA91576 | 77 | alpha-tubulin |
| brain | 45 | AAH12854 | 127 | actin |
| testis | 50 | AAC39578+EAW88369 | 275 | alpha-tubulin+beta-tubulin |
| kidney | 50 | AAC39578+ EAW88369 | 289 | alpha-tubulin+ beta-tubulin |
| liver | 50 | CAA25855+NP_006079 | 157 | alpha-tubulin+ beta-tubulin |
