# Supplementary figures and images for: YB-1 promotes microtubule assembly in vitro through interaction with tubulin and microtubules
Source: BMC Biochem. 2008 Sep 15;9:23. doi: 10.1186/1471-2091-9-23 (PMC2557009; doi:10.1186/1471-2091-9-23)

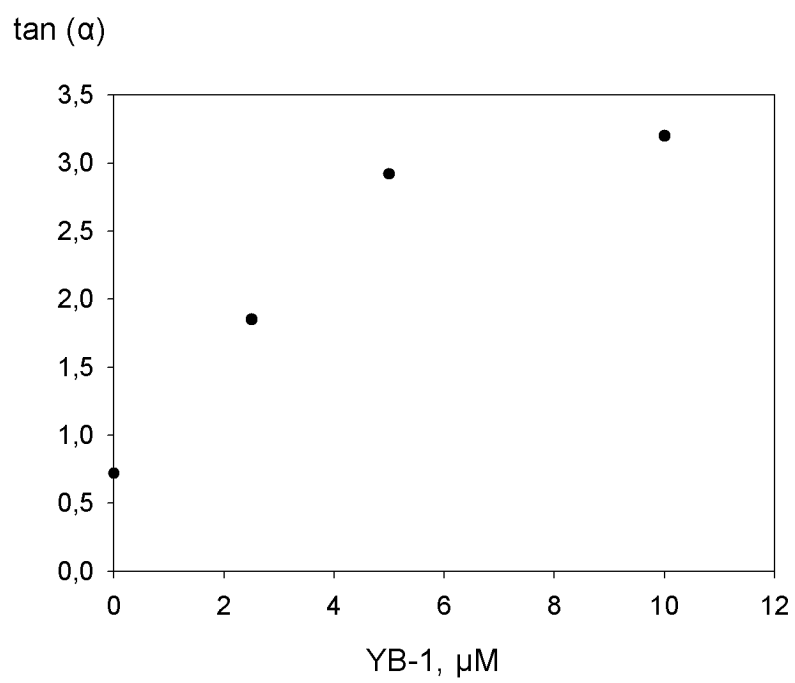

Supplement: Additional file 4 — Plot of the tangent at the microtubule assembly slope versus YB-1 concentration observed on figure 5. We can notice that rate of microtubule assembly reaches a maximum plateau value from about 8 μM YB-1. [file 1471-2091-9-23-S4.pdf]

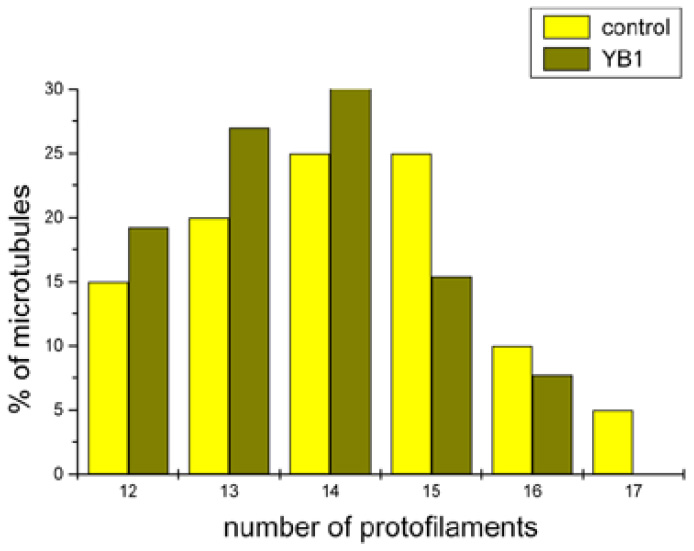

Supplement: Additional file 2 — Histogram of protofilament number observed in control microtubules and in the presence of YB-1. [file 1471-2091-9-23-S2.jpeg]

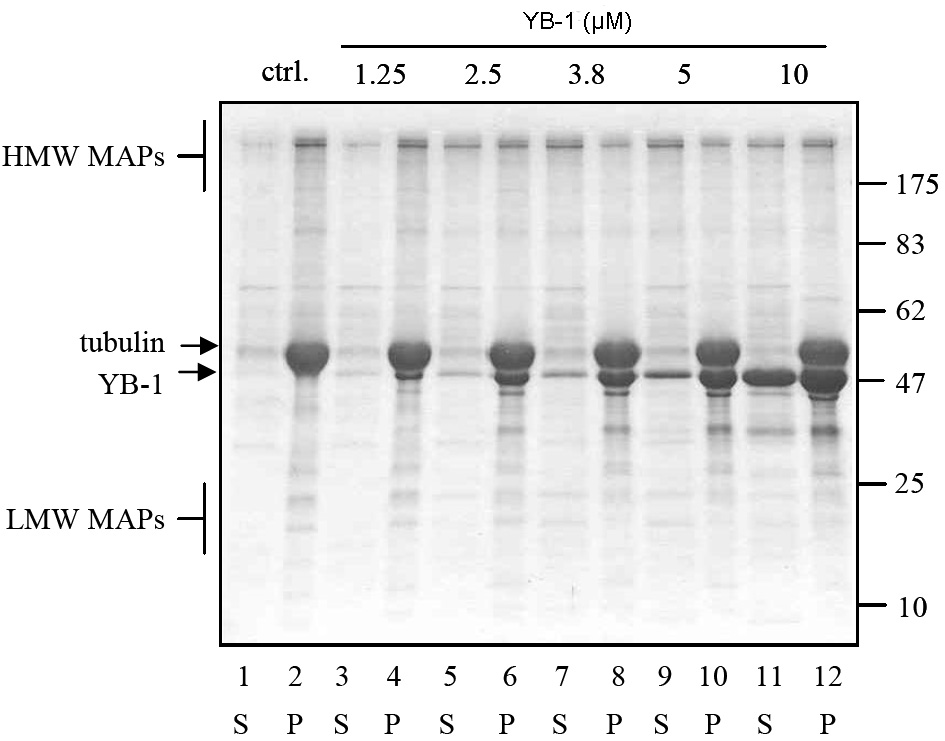

Supplement: Additional file 3 — YB-1 partially displaces MAPs from taxol-stabilized microtubules. MAPs-tubulin (0.5 mg/ml, ~4μM) was polymerized in the absence (control) or presence of increasing concentrations of YB-1 (from 1.25 μM to 10 μM, as indicated) in buffer M with 10% glycerol and 20 μM taxol. After polymerization, the samples were pelleted, and equal volumes of supernatants and resuspended pellets were analyzed by SDS-PAGE. (S, supernatant; P, pellet). [file 1471-2091-9-23-S3.jpeg]
